# Evaluation of SARS-CoV-2 anti-Spike antibody levels and breakthrough infection risk among vaccinated adults in North Lebanon

**DOI:** 10.1371/journal.pone.0302579

**Published:** 2024-05-09

**Authors:** Dalal Nour, Mohamad Bachar Ismail, Marwan Osman, Rayane Rafei, Dalal Kasir, Fouad Dabboussi, Philippe Colson, Monzer Hamze

**Affiliations:** 1 Laboratoire Microbiologie Santé et Environnement (LMSE), Doctoral School of Science & Technology, Faculty of Public Health, Lebanese University, Tripoli, Lebanon; 2 Aix-Marseille Univ., Institut de Recherche pour le Développement (IRD), Microbes Evolution Phylogeny and Infections (MEPHI), Marseille, France; 3 Faculty of Sciences, Lebanese University, Tripoli, Lebanon; 4 Department of Neurosurgery, Yale University School of Medicine, New Haven, CT, United States of America; 5 IHU Méditerranée Infection, Marseille, France; 6 Assistance Publique-Hôpitaux de Marseille (AP-HM), Marseille, France; Sheikh Hasina National Institute of Burn & Plastic Surgery, BANGLADESH

## Abstract

Since March 2020, the COVID-19 pandemic has swiftly propagated, triggering a competitive race among medical firms to forge vaccines that thwart the infection. Lebanon initiated its vaccination campaign on February 14, 2021. Despite numerous studies conducted to elucidate the characteristics of immune responses elicited by vaccination, the topic remains unclear. Here, we aimed to track the progression of anti-spike SARS-CoV-2 antibody titers at two-time points (T1: shortly after the second vaccination dose, T2: six months later) within a cohort of 201 adults who received Pfizer-BioNTech (BNT162b2), AstraZeneca, or Sputnik V vaccines in North Lebanon. Blood specimens were obtained from participants, and antibody titers against SARS-CoV-2 were quantified through the Elecsys-Anti-SARS-CoV-2 S assay (Roche Diagnostics, Switzerland). We used univariate analysis and multivariable logistic regression models to predict determinants influencing the decline in immune response and the occurrence of breakthrough infections among vaccinated patients. Among the 201 participants, 141 exhibited unchanging levels of antibody titers between the two sample collections, 55 displayed waning antibody titers, and only five participants demonstrated heightened antibody levels. Notably, age emerged as the sole variable significantly linked to the waning immune response. Moreover, the BNT162b2 vaccine exhibited significantly higher efficacy concerning the occurrence of breakthrough infections when compared with the AstraZeneca vaccine. Overall, our study reflected the immune status of a sample of vaccinated adults in North Lebanon. Further studies on a larger scale are needed at the national level to follow the immune response after vaccination, especially after the addition of the third vaccination dose.

## Introduction

In December 2019, an outbreak of atypical pneumonia was reported in Wuhan City, China [1]. Its rapid spread across the country and around the world led the World Health Organization (WHO), in March 2020, to identify this new infectious disease of Corona Virus (COVID-19), a global pandemic [2]. As of 22 November 2023, the Coronavirus disease (COVID-19), caused by the Severe Acute Respiratory Syndrome Corona Virus 2 (SARS-CoV-2), has attacked more than 772 million people and its-related cumulative deaths have exceeded 6.9 million around the world [3]. The genome codes for 4 structural proteins: Spike protein (S), Nucleocapsid (N), Membrane (M), and Envelope (E), and 16 non-structural proteins noted (nsp1-16) [4]. This virus enters the target cell after binding its Receptor Binding domain (RBD), present on the S1 subunit of S glycoprotein, with Angiotensin-Converting Enzyme 2 (ACE 2) receptor present on the surface of epithelial cells of the nasal cavity, to begin its viral replication. SARS-CoV-2 triggers the production of host antibodies that begins early against the N protein, while it takes 4–8 days after symptom onset to release antibodies against the S protein [5, 6].

Different types of COVID-19 vaccines have been designed. The first vaccine that has been FDA-approved for people above 18 years was Pfizer-BioNTech (BNT162b2) (now called Comirnaty). It is an mRNA-based vaccine surrounded by lipid nanoparticles and coding for the full-length spike protein of SARS-CoV-2, stimulating the immune response of the body against the virus [7, 8]. AstraZeneca-vaccine (ChAdOx1 nCoV-19), known by Vaxzevria and Covishield as trademarks, is a viral vector vaccine coding for a full-length S-Protein of SARS-CoV-2 [9, 10]. It is given also to individuals above 18 years old [11]. Sputnik V (Gam-COVID-Vac) vaccine is another example of a viral vector vaccine that encodes a full-length S-protein of SARS-CoV2 [12, 13]. As of 18 November 2023, more than 13,5 billion COVID-19 vaccines have been administered around the world according to the WHO [3].

The antibody levels developed against SARS-CoV-2 after infection or after vaccine intake can be measured by serological tests, based on the specific binding between antigen and antibodies [14]. Different methods can be used to detect anti-SARS-CoV-2 antibodies including the chemiluminescence immunoassay (CLIA) which represents a high-throughput assay that can test a large number of samples at the same time with accurate results and low signal-to-noise ratio [15, 16]. Elecsys^®^ Anti-SARS-CoV-2 S developed by Roche, is one of the CLIA immunoassays that detects and quantifies total antibody titers of high affinity to SARS-CoV-2 S protein RBD with high sensitivity and specificity [17].

Lebanon started its vaccination campaign against COVID-19 on Sunday, February 14, 2021, using the BNT162b2 vaccine [18]. However, a notable lack of data prevails regarding SARS-CoV-2 antibody dynamics and the comparative efficacy of distinct vaccine types in Lebanon. To address this knowledge gap, this study aimed to monitor the level of anti-Spike SARS-CoV-2 antibody titers and breakthrough infections among a demographically diverse adult vaccinated population at two time points (referred to as T1 and T2), after the second booster vaccination dose. Notably, the detection of anti-Spike SARS-CoV-2 antibody titers was performed through the Elecsys-anti-SARS-CoV-2 S assay.

## Materials and methods

### Ethics statement

The study was conducted in accordance with the Declaration of Helsinki and approved by the institutional review board (IRB) of the Nini Hospital (IRB-F01-29.3.2021; March 29, 2021). Written informed consent was obtained from all subjects involved in the study.

### Study design

Initially, BNT162b2 emerged as Lebanon’s only available COVID-19 vaccine, with a primary focus on safeguarding the elderly (>75 years) and healthcare professionals [19]. In this context, our recruitment efforts started with these two pivotal groups from various regions in North Lebanon, each of whom had received the BNT162b2 vaccination. Additional vaccine options were later available, including AstraZeneca and Sputnik V. Subsequently, our study population expanded to include a broader demographic, encompassing individuals of all ages above 18 from the same geographical area. The inclusion criteria for participation in the study include: Being aged 18 or above, having completed the full regimen of a singular COVID-19 vaccine type, with the second dose administered from 14 days to 3 months before the collection of blood samples for the study. Moreover, individuals were required to have no prior history of COVID-19 infection or any indicative symptoms.

### Sampling and data collection

**Fig 1:**
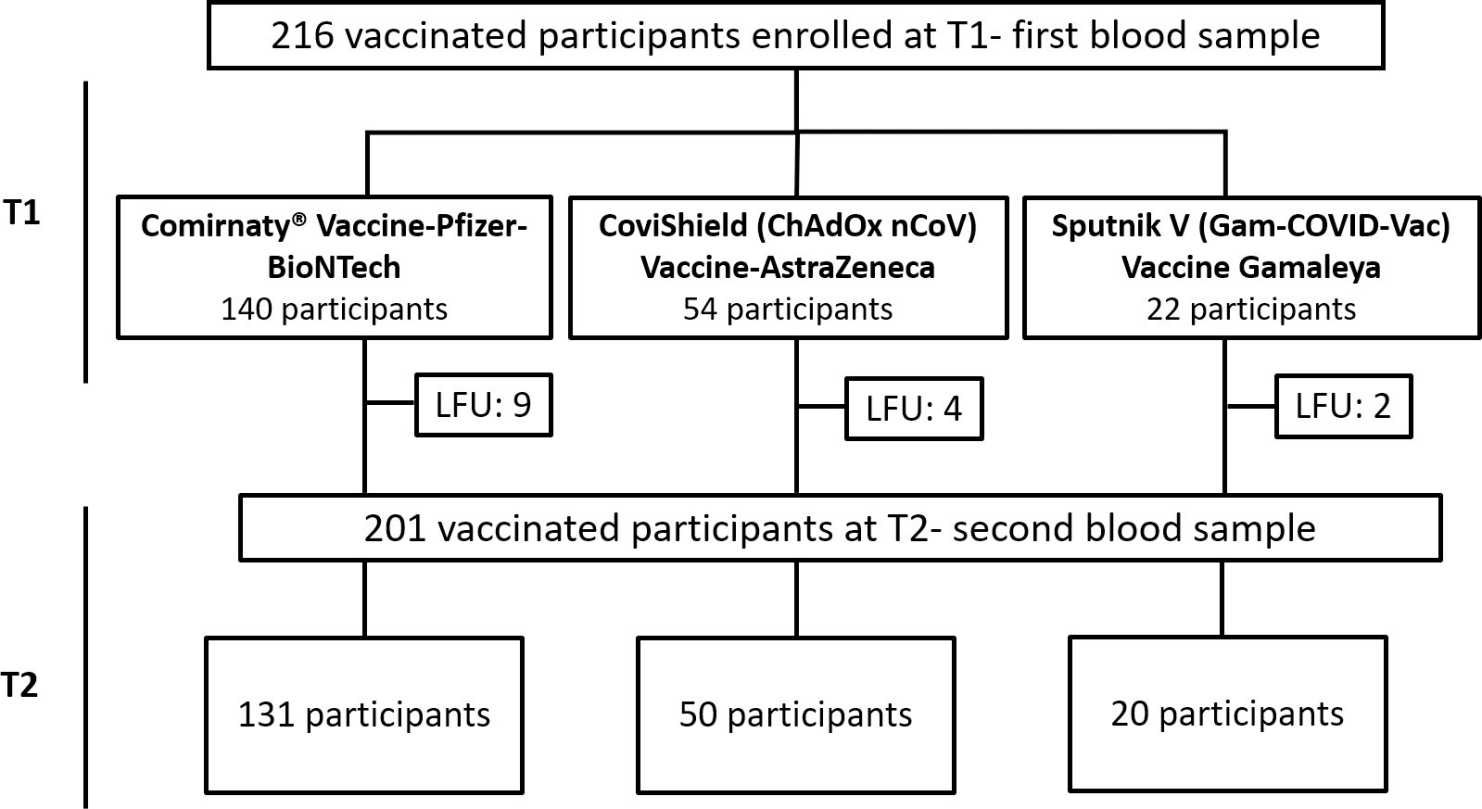
Flowchart showing the number of participants enrolled in the study based on the type of vaccine taken (Comirnaty^®^ vaccine-BNT162b2, CoviShield (ChAdOx1 nCoV) vaccine-AstraZeneca, Sputnik V(Gam-COVID-Vac) vaccine-Gamaleya). LFU: Loss to follow-up.

After a signed consent for the participation in the study, demographic, socio-economic, health, and vaccine-related data were collected from each eligible participant, using an online questionnaire. Afterwards, 2 blood samples were collected: the first one was collected from 14 to 30 days after taking the second dose of the vaccine (T1) and the second one was taken 6 months after (T2). The sampling was performed between April 1, 2021, and March 31, 2022. In total, we have recruited 216 participants: 140 have received the BNT162b2 vaccine, 54 have received the AstraZeneca vaccine, and 22 have received the Sputnik V vaccine. However, 15 cases were lost of follow-up, and thus, second blood samples were obtained from 201 participants. The number of participants enrolled in the study is shown in the following flowchart, based on the type of vaccine taken (Fig 1).

### Elecsys^®^ Anti-SARS-CoV-2 S assay

Anti-spike antibody titers were assessed at T1 and T2 using the Roche Elecsys® Anti-SARS-CoV-2 S test on the cobas™ e801 (Roche Diagnostics, Mannheim, Germany). The assay and results interpretation were conducted according to the manufacturer’s instructions: results <0.80 U/ml were considered negative for anti-SARS-CoV-2-S while those = or > 0.80 U/ml were considered positive. Results that exceed a value of 250 U/ml were recorded as > 250 U/ml. To note, the unit “U/ml” obtained in Elecsys^®^ anti-SARS-CoV-2 S assay results is equivalent to WHO BAU/ml [17]. Comparative analysis was then done using Microsoft Excel software to determine the evolution of anti-Spike SARS-CoV-2 antibody titers between T1 and T2 in each participant.

### Measuring breakthrough infections

After taking the second blood sample, participants were asked if they had developed COVID-19 symptoms and whether they had done a PCR test confirming an infection with SARS-CoV-2 in the period between the first (T1) and the second (T2) sampling, searching for any breakthrough infection after they have completed their vaccine regimen.

### Statistical analysis

Data recorded in the questionnaire and the laboratory analysis were evaluated for completeness and consistency before analysis using the R software (R Core Team, version 4.2.2; R Studio, version 2023.06.1+524 "Mountain Hydrangea"). The dataset was imported for cleaning, variable coding, and subsequent analysis. Descriptive analysis was performed on all variables using several packages (e.g., dplyr, stringr, prettyR, summarytools). Data were presented as mean [standard deviation (SD); min-max] for continuous variables and as frequency distributions for categorical variables. To predict the determinants of a decrease in COVID-19 immunity among vaccinated patients over 6 months at the univariate level, we compared the differences across groups using the t-test and the Pearson chi-squared test for continuous and categorical variables, respectively. The decrease in COVID-19 immunity (yes/no) and the occurrence of a breakthrough infection among vaccinated patients (yes/no) were the outcomes and sociodemographic, behavioral, and clinical data were the explanatory variables. Subsequently, multivariable logistic regression analysis was done, including variables with a P-value (*P*) < 0.40 obtained at the univariate level. We also performed a backward stepwise model to identify and confirm the associations of covariates with the decrease in COVID-19 immunity. Statistical tests were two-sided, with a type I error set at α =  0.05.

## Results

### Characteristics of the population

**Fig 2:**
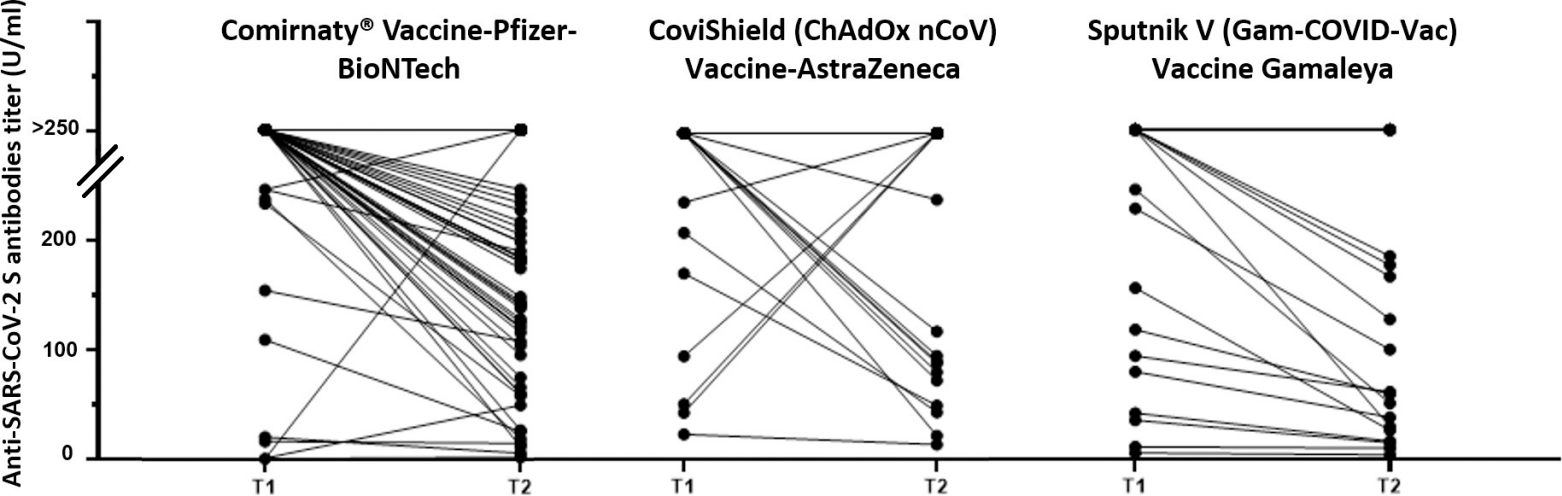
Levels of Anti-SARS-CoV-2 S antibody titers (U/ml) measured during two time points (T1 and T2). T1 ranges between 2 weeks and 2 months after receiving the COVID-19 booster dose. T2 represents a 6-month duration after the initial collection. These measurements were taken from participants who had received the Pfizer-BioNTech (BNT162b2), AstraZeneca, or Sputnik V vaccine. ^*//*^The upper values (>200 U/ml) do not follow the same scale as the lower ones (≤ 200 U/ml).

After excluding 15 individuals who were lost of follow-up from the rest of the statistical analysis, the total number of the population studied was 201 individuals. 131 were vaccinated with BNT162b2, 50 with AstraZeneca, and 20 with Sputnik V vaccines. As a data summary, 48.8% of the participants were women, and 51.2% were men. The mean age of the participants was 51.7 years (standard deviation = 16.6; min-max = [21–91]). Regarding smoking and alcoholism status, 39.8% were smokers and 13.4% were alcohol consumers. Data regarding body Mass Index (BMI), blood type, the presence of any chronic disease, and the symptoms developed after vaccine intake were also collected from participants. Anti-Spike SARS-CoV-2 antibody titers were stratified as follows: high (>249 U/ml), moderate (100–249 U/ml) and low (<100 U/ml). This stratification was based on previous studies having used Elecsys^®^ Anti-SARS-CoV-2 S assay [20–23] (Table 1). Although we monitored the antibody levels (Fig 2), we assessed changes in the immune response between the initial and subsequent samplings by comparing them to the predefined levels of immune response (Table 1). To clarify, if the antibody titer transitions from the high to the moderate level, it indicates a decrease in the immune response. Additionally, maintaining the same level of antibody titer (i.e., high, moderate, low) regardless of the antibody titer value between the first and second samplings is considered indicative of stability.

**Table 1:** Sociodemographic and clinical characteristics of the study population, stratified by the temporal trend of COVID-19 antibodies.

	Total n = 201		Decrease in antibodies’ trend n = 55		Stability in antibodies’ trend n = 141		Increase in antibodies’ trend n = 5	
	n	%	n	%	n	%	n	%
**Antibodies’ level (U/ml)** **(T**_**1**_^**¶**^ **)**								
High (>249)	**176**	87.6	48	87.3	128	90.8	0	0.0
Moderate (100–249)	**10**	5.0	7	12.7	2	1.4	1	20.0
vLow (<100)	**15**	7.5	0	0.0	11	7.8	4	80.0
**Antibodies’ level (U/ml)** **(T**_**2**_ **= 6 months)**								
High (>249)	**133**	66.2	0	0.0	128	90.8	5	100.0
Moderate (100–249)	**35**	17.4	33	60.0	2	1.4	0	0.0
Low (<100)	**33**	16.4	22	40.0	11	7.8	0	0.0
**Sex**								
Female	**98**	48.8	27	49.1	68	48.2	3	60.0
Male	**103**	51.2	28	50.9	73	51.8	2	40.0
**Age (years)**								
<50	**99**	49.2	21	38.2	75	53.2	3	60.0
[50–75]	**82**	40.8	26	47.3	54	38.3	2	40.0
≥75	**20**	10	8	14.5	12	8.5	0	0.0
**Smoking**								
No	**121**	60.2	35	63.6	85	60.3	1	20.0
Yes	**80**	39.8	20	36.4	56	39.7	4	80.0
**Alcohol consumption**								
No	**174**	86.6	45	81.8	124	87.9	5	100.0
Yes	**27**	13.4	10	18.2	17	12.1	0	0.0
**Body Mass Index (kg/m** ^**2**^ **)** **(Mean [SD; min-max])**	27.1 [5.8; 17.6–67.6]		27.3 [5.3; 19.1–46.9]		26.9 [6; 17.6–67.6]		29.2 [5.7; 21.5–35.5]	
**Blood type & Rhesus factor**								
Unknown	**15**	7.5	4	7.3	10	7.1	1	20.0
A^-^	**7**	3.5	1	1.8	6	4.3	0	0.00
A^+^	**57**	28.4	13	23.6	42	29.8	2	40.0
AB^+^	**10**	5.0	4	7.3	6	4.3	0	0.0
B^-^	**6**	3.0	2	3.6	4	2.8	0	0.0
B^+^	**29**	14.4	6	10.9	22	15.6	1	20.0
O^-^	**9**	4.5	3	5.5	5	3.5	1	20.0
O^+^	**68**	33.8	22	40.0	46	32.6	0	0.0
**Chronic disease**								
No	**112**	55.7	29	52.7	82	58.2	1	20.0
Yes	**89**	44.3	26	47.3	59	41.8	4	80.0
**Vaccine name**								
Pfizer-BioNTech (BNT162b2)	**131**	65.2	37	67.3	93	66.0	1	20.0
AstraZeneca	**50**	24.9	10	18.2	36	25.5	4	80.0
Sputnik V	**20**	10.0	8	14.5	12	8.5	0	0.0
**Side effects after the first shot**								
No	**57**	28.4	18	32.7	39	27.7	0	0.0
Yes	**144**	71.6	37	67.3	102	72.3	5	100.0
**Side effect or symptoms after second shot**								
No	**62**	30.8	21	38.2	40	28.4	1	20.0
Yes	**139**	69.2	34	61.8	101	71.6	4	80.0
**Breakthrough cases** *								
No	**193**	96	54	98.2	135	95.7	4	80.0
Yes	**8**	4	1	1.8	6	4.3	1	20.0

*During the study period. ¶T1 ranges between 2 weeks and 2 months after receiving the COVID-19 booster dose; T2 represents a 6-month duration after the initial collection.

### Evolution of SARS-CoV-2 anti-S antibody titers

Our results show that 55 of the participants (27.4%) confronted a decrease in the antibody titer between the first and second sampling, among them, 37 (67.3%) were vaccinated with BNT162b2, 10 have taken AstraZeneca (18.2%) and 8 were vaccinated with Sputnik V (14.6%). Only 5 participants have shown an increase in the antibody titers, among them 4 were vaccinated with AstraZeneca and 1 with BNT162b2. Stability in the antibody titers was detected in the remaining 141 participants (70.1%), with 90.8% among them maintaining a high antibody level. Notably, no negative results (i.e., SARS-CoV-2 anti-S antibody titer < 0.8 U/ml) have been detected neither in T1 nor in T2 results. The levels of anti-Spike SARS-CoV-2 antibody at both T1 and T2 and their evolution between these two-time points are shown in Fig 2.

Age was the only variable significantly associated with the decrease in immune levels (Table 2). The mean age of people who showed a significant decrease in SARS-CoV-2 anti-S antibody titers was 56.3 years (standard deviation = 16.0; min-max = [24–87]).

**Table 2:** Determinants of decrease in COVID-19 immunity among vaccinated patients over a 6-month period using univariate analysis and multivariable logistic regression models.

	Univariate analysis		Multivariable logistic regression models						
			**Model 1** ^**i**^			**Model 2** ^**ii**^			
***Categorical variables***	%	***P***	**adj. OR**	**95%CI**	***P***	**adj. OR**	**95%CI**	***P***	
**Sex**									
Female^1^	28.4								
Male	27.8	1.00							
**Smoking**									
No^1^	29.2								
Yes	26.3	0.79							
**Alcohol consumption**									
No^1^	26.7								
Yes	37.0	0.37	1.67	0.67–3.99	0.26				
**Blood type & Rhesus factor**									
A^-1^	28.6								
A^+^	14.3								
AB^+^	40.0								
B^-^	33.3								
B^+^	21.4								
O^-^	37.5								
O^+^	32.4	0.82							
**Chronic disease**									
No^1^	26.1								
Yes	30.6	0.60							
**Vaccine type**									
Pfizer-BioNTech (BNT162b2)^1^	28.5								
AstraZeneca	21.7		0.69	0.29–1.54	0.38				
Sputnik V	40.0	0.31	1.95	0.69–5.32	0.19				
**Side effects after the first dose**									
No^1^	31.6								
Yes	26.6	0.60							
**Side effects after the booster dose**									
No^1^	34.4								
Yes	25.2	0.25	0.75	0.38–1.53	0.42				
**Breakthrough cases** *									
No^1^	28.6								
Yes	14.3	0.68							
***Continuous variables***	*Decrease in antibodies’ level*	*No decrease in antibodies’ level*	***P***	**adj. OR**	**95%CI**	***P***	**adj. OR**	**95%CI**	***P***
**Age**	**56.3**	**49.9**	**0.02**	**1.02**	**1.00–1.04**	**0.04**	**1.02**	**1.00–1.04**	**0.02**
**Body Mass Index (BMI)**	27.3	26.9	0.62						

^¶^Determinants of decrease in COVID-19 immunity were predicted using univariate (t-test and the chi-squared test for continuous and categorical variables, respectively) and multivariable analysis (logistic regression models). ^i^The variables tested by univariate analysis that had a P-value (*P*) < 0.40 were included in Model 1 (multivariable logistic regression analysis). ^ii^In Model 2, a backward logistic regression model was created including only complete cases. ^1^Reference group. *During the study period.

### Determinants of breakthrough infection

In total, among the 201 participants vaccinated with 2 doses, 29 reported COVID-19 symptoms between the first and second sampling time, and 16 among them underwent PCR leading to the confirmation of 8 breakthrough infections. Results are shown in Table 3 according to the 3 types of vaccine. Overall, eight individuals developed COVID-19-like symptoms over the study period and received a positive PCR result. The mean age of these participants was 51.4 years, with six and two being women and men, respectively. Moreover, five of them suffer from clinical comorbidities. Notably, although five of these cases suffered from comorbidities making them at risk for severe COVID-19, symptoms were moderate, and nobody developed severe disease.

**Table 3:** Number of participants developing COVID-19 symptoms between T1 and T2 and whether this was confirmed or not by a PCR test.

Vaccine type	Participants with COVID-19 symptoms	Symptomatic participants tested by PCR	Unconfirmed infection (negative or indeterminate PCR)	Confirmed infection (positive PCR)
Pfizer-BioNTech (BNT162b2)	14	9	6	3
AstraZeneca	12	6	1	5
Sputnik	3	1	1	-
Total	29	16	8	8

After predicting the determinants of breakthrough infection with SARS-CoV-2 among the participants over the 6 months, the type of vaccine AstraZeneca was the only variable associated significantly with the appearance of breakthrough infections (Table 4). This means that the BNT162b2 vaccine has shown more effectiveness than the AstraZeneca vaccine about this parameter. Individuals who were vaccinated with Sputnik V were removed from this analysis because of their low total number.

**Table 4:** Determinants of breakthrough infections among vaccinated patients over a 6-month period using univariate analysis and multivariable logistic regression models.

	Univariate analysis		Multivariable logistic regression models						
			**Model 1** ^**i**^			**Model 2** ^**ii**^			
***Categorical variables***	%	***P***	**adj. OR**	**95%CI**	***P***	**adj. OR**	**95%CI**	***P***	
**Sex**									
Female^1^	6.81								
Male	2.15	0.16	0.35	0.05–1.63	0.21	0.30	0.04–1.37	0.15	
**Smoking**									
No^1^	3.57								
Yes	5.80	0.48							
**Alcohol consumption**									
No^1^	5.09								
Yes	0.00	0.60							
**Blood type & Rhesus factor**									
A^-1^	0.00								
A^+^	3.92								
AB^+^	10.0								
B^-^	0.00								
B^+^	11.5								
O^-^	0.00								
O^+^	3.33	0.59							
**Chronic disease**									
No^1^	2.91								
Yes	6.41	0.29	2.48	0.55–13.1	0.25				
**Vaccine type** ^**¶**^									
Pfizer-BioNTech (BNT162b2)^1^	**2.29**								
AstraZeneca	**10.0**	**0.04**	**5.35**	**1.22–27.9**	**0.03**	**4.75**	**1.11–24.2**	**0.04**	
**Side effects after the first shot**									
No^1^	4.00								
Yes	4.58	1.00							
***Continuous variables***	*Breakthrough infection*	*No breakthrough infection*	***P***	**adj. OR**	**95%CI**	***P***	**adj. OR**	**95%CI**	***P***
**Age (years)**	51.4	52.2	0.86						
**Body Mass Index (kg/m** ^**2**^ **)**	28.1	27.1	0.74						

^¶^Determinants of breakthrough infections were predicted using univariate (t-test and the chi-squared test for continuous and categorical variables, respectively) and multivariable analysis (logistic regression models). ^i^The variables tested by univariate analysis that had a P value (*P*) < 0.40 were included in Model 1 (multivariable logistic regression analysis). ^ii^In Model 2, a backward logistic regression model was created including only complete cases. ^1^Reference group. *During the study period. ^¶^Individuals who had received a Sputnik V vaccination were removed from the analysis because of their low total number.

## Discussion

This study enrolled 201 individuals who were 2-dose vaccinated with one of these 3 types of vaccines: BNT162b2, AstraZeneca, or Sputnik V, in North Lebanon. Enrolled participants were not previously infected by SARS-CoV-2, meaning that their immune response is mainly induced by vaccination intake. SARS-CoV-2 antibody titers against Spike protein were evaluated in 2 different time points (T1 shortly after the second vaccine dose and T2, 6 months after), by Elecsys-Anti-SARS-CoV- 2 S assay, an accurate, highly specific, and sensitive CLIA assay. The 3 levels of stratification, high (>249 U/ml), moderate (100–249 U/ml), and low (<100 U/ml), for the anti-Spike SARS-CoV-2 antibody titers in our study were based on previous studies [20–23].

In our study, the majority (70.14%) of the participants have shown stability in the antibody titers between the first and second sampling, while a decrease in the immune response was observed in 27.4% of the participants. An increase in antibody titers was observed in 5 participants. In all of those 3 cases, antibody presence was still detectable after 6 months after the booster dose intake of the vaccines. Regarding the correlation between variables and the decrease in immune responses, our study shows that only age was significantly associated with this decrease.

This result was unsurprising because age is associated with the decline and dysregulation of many immune mechanisms, resulting in a deficit in both innate and adaptive immune responses [24]. A validation prospective, longitudinal cohort study was carried out by Lustig *et al*., who followed the immune responses of 2607 healthcare workers who tested negative for anti-SARS-CoV-2 IgG before receiving the first dose of BNT162b2 vaccine. The follow-up was done 3 times: at 1–2 weeks after the first vaccine dose, at the time of the second dose administration, and at 1–2 weeks after the second dose. Results of this study showed that male sex, age, and comorbidities were associated with lower antibody levels [25]. Another retrospective cohort in the United States including a large number of individuals above 12 years (approximately 3.5 million people), followed the effectiveness of the BNT162b2 vaccine over time for up to 6 months after taking the vaccination. A decline in the vaccine effectiveness against SARS-CoV-2 infection has been recorded between the first month after full vaccination (88% of effectiveness (95% CI: 86–89)) and after 5 months (47% of effectiveness (95% CI:43–51)). Vaccine effectiveness in individuals older than 65 years declined from 80% (95% CI: 73–58; first month) to 43% (95%CI: 30–54) [26]. In the same context, IgG antibody levels were followed up in a prospective longitudinal study 4 times after BNT162b2 vaccination: S1: 3 weeks after the first vaccine dose, S2: 1 month after the second dose, S3: 3 months after the second dose and S4: 6 months after the second dose [27]. Participants in this study had or did not have a pre-vaccination history of COVID-19 infection. A significant difference in antibody titers was detected between age groups; participants aged between 18 and 30 showed the highest antibody levels at all of the sampling times, while the lowest antibody titers were detected in participants older than 60 years at S1, S2, and S4, which is in concordance with our study [27]. However, these differences seem to decrease with time, were more accentuated at S1 sampling time, and lowered significantly after 6 months of the second booster dose. Nonetheless, this analysis did not include participants older than 66 years, who may develop lower antibody titers after the first and possibly the booster dose as well [27]. With regards to COVID-19 pre-vaccination status, a moderate negative correlation between age and antibody levels was detected at S1, and this correlation decreased with time for naïve COVID-19 participants. Individuals, who were previously infected with COVID-19, developed higher antibody levels at S1, S3, and S4 regardless of their age [27]. Moreover, a cohort study by Müller *et al*. comparing the immune response after the first and second dose of the BNT162b2 vaccine found that antibody titers were significantly higher in the group of younger participants (below 60 years) compared to elderly ones (over 80 years) [28].

On the other hand, a similar study conducted in Poland investigated the levels of anti-SARS-CoV-2 S antibodies in a population being infected or not with SARS-CoV-2, 8 months after taking the second dose of the BNT162b2 vaccine [29]. The results showed that convalescent participants developed a significant increase in the immune response that was associated with age, severity of COVID-19 symptoms, and their work type. In contrast to our results, the authors found that participants older than 50 years showed also higher levels of anti-SARS-CoV-2 S antibodies after the BNT162b2 vaccine intake in the group of participants non-previously infected with COVID-19, but this result was not statistically significant [29]. Furthermore, Subbarao *et al*. studied a population of adults aged 70 years and above vaccinated with the BNT162b2 vaccine, showing that anti-S SARS-CoV-2 antibody levels after 2 doses of the vaccine were very robust, significantly higher than levels of antibodies in convalescent participants previously infected with COVID-19 [30]. While, in the study of Tretyn *et al*. on individuals vaccinated by the BNT162b2 vaccine, no significant association was observed between age and anti-SARS-CoV-2 IgG antibody concentrations [31].

Further, our study has investigated the determinants of breakthrough infection in participants after 6 months of the booster vaccination dose. While Sputnik V vaccinated individuals were excluded from this analysis because of their low headcount, our data showed that the AstraZeneca vaccine was more associated with breakthrough infection than the BNT162b2 vaccine. Notably, our study was conducted from April 2021 to March 2022, a time during which Lebanon experienced successive domination by the Alpha, Delta, and Omicron variants [32]. We observed four breakthrough infection cases in November 2021, when the Delta variant was the predominant strain. The remaining four breakthrough infection cases occurred between December 2021 and January 2022, coinciding with the period dominated by the Omicron variant [32]. This observation may provide additional insights into the potential association between breakthrough infections and the Delta and Omicron variants. Indeed, although some data revealed that 2 doses of the BNT162b2 or AstraZeneca vaccines have the same effectiveness against SARS-CoV-2 Alpha and Delta variants [33], other studies reported that individuals vaccinated with BNT162b2 are more protected than those vaccinated with AstraZeneca. In this context, an observational study, conducted in the United Kingdom, examined the effectiveness of 2-dose vaccines of either BNT162b2 or AstraZeneca, against infection with Alpha or Delta variants of SARS-CoV-2. Results showed that even though the effectiveness was reduced in Delta variant infection for both vaccines, the BNT162b2 vaccine has shown higher effectiveness for the 2 variants: (93.7% (95%CI: 91.6–95.3) for Alpha, and 88% (95%CI: 85.3–90.1) for Delta variant. However, the effectiveness of the AstraZeneca vaccine was 74.5% (95% CI: 68.4–79.4) for the Alpha variant and 67% (95%CI: 61.3–71.8) for Delta [34]. Besides, Stouten *et al*. showed that the 2 viral-vector vaccines JNJ-78436735 (Ad26.COV2.S) and AstraZeneca vaccine (ChAdOx1 nCoV-19) were associated with a higher risk of breakthrough infection compared to BNT162b2 [35]. This is concordant with a study conducted in Iraq, showing that the AstraZeneca vaccine was more associated with breakthrough infections in comparison with mixed vaccines or BNT162b2 vaccines [36]. A study in the United Kingdom including a group of vaccinated index patients and unvaccinated ones, compared the probability of infection of people in contact with these 2 groups. It showed that people vaccinated with BNT162b2 were at a lower probability of infecting their contacts than AstraZeneca vaccinated index patients, making BNT162b2 more protective than AstraZeneca [37, 38]. 192 individuals having received 2 doses of either BTN162b2 or AstraZeneca vaccine and a booster third dose of BTN162b2 vaccine were followed up in an investigation conducted in Poland, using Elecsys-anti-SARS-CoV-2 S to measure antibody levels. Total antibody titers were significantly higher in participants who have received 3 doses of BTN162b2 than those who have received 2 doses of AstraZeneca and a booster dose of BTN162b2 [39].

Moreover, previous studies have shown a significant increase in breakthrough infection with the apparition of a new variant of concern compared with prior strains circulating [40]. Indeed, the study of Kustin *et al*. in people vaccinated with BNT162b2 revealed more breakthrough infection cases with the apparition of the Alpha variant compared with previous strains [41]. Equally, a study in the United States investigated the effectiveness of BNT162b2, mRNA-1273, and Janssen Ad26.COV2.S vaccines, showing that more breakthrough infections were documented with the emergence of the Delta variant [42]. Furthermore, a study conducted in Germany investigated SARS-CoV-2 breakthrough infection in healthcare workers fully vaccinated (3 vaccine doses) with one of these types of vaccines (BNT162b2, Vaxzevria (viral vector vaccine), Spikevax (mRNA vaccine) or Jcovden (viral vector vaccine)), have noticed that the number of breakthrough infections has increased rapidly subsequently with the regional spread of Delta and Omicron variants [43]. Additionally, an investigation in South Africa describing breakthrough infections after vaccination in the period of domination of Delta and Omicron variants has shown that these infections were more than 3 times higher when Omicron was dominating than at the peak of the Delta variant domination [44]. A recent case-control analysis on breakthrough infections in the periods of the emergence of Delta and Omicron variants in New York has shown that the Omicron variant was associated with a higher level of breakthrough in fully vaccinated and boosted participants. The vaccines that were taken in this study were Moderna, BNT162b2, and Janssen [40]. On the other hand, Regenhardt *et al*. found that Delta and Omicron breakthrough infections were associated with higher viral load than breakthrough infections reported with the Alpha variant, this may be associated with the immune evasive characteristics of these variants [43, 45]. This is concordant with our study, where all the breakthrough infection cases recorded an antibody titer higher than 250 U/ml at the second sampling and were all observed in the period of the domination of the Delta and Omicron variants in Lebanon.

Finally, it should be mentioned that like any epidemiological survey, our study is not without limitations, including mainly the low number of participants. Besides, we were not able to do dilutions for samples having an antibody titer >250 U/ml and remeasure anti-Spike antibody titers in order to get the exact concentration. Indeed, during the period of this study, Lebanon faced a catastrophic economic crisis and the currency has lost more than 95 percent of its pre-crisis value, thus limiting our ability to purchase additional assay kits. This step would be important to compare the vaccine effectiveness regarding the level of the immune response. Finally, the Sputnik V vaccine was not included in the further analysis in reason to its low headcount.

## Conclusions

This study investigated SARS-CoV-2 anti-S antibody titers in a population of 201 individuals vaccinated with two doses against SARS-CoV-2 in North Lebanon. The measurement was accomplished 2 times (T1, 14 to 30 days after the booster dose, and T2, 6 months after). The 3 vaccines examined in the study were: BNT162b2, AstraZeneca, and Sputnik V. The results show that 70.14% of the participants maintained their immune response between the 2 sampling, 27.4% manifested a decrease in the antibody titers, while only 2.5% showed an increase in the antibody levels. Age was the only variable significantly associated with the decrease in the immune response. On the other hand, participants vaccinated with BNT162b2 manifested fewer breakthrough infections than those vaccinated with AstraZeneca.

Overall, our study has provided an overview of the evolution of the immune response after COVID-19 vaccination in a small sample of demographically diverse vaccinated adults in North Lebanon. Notably, after the addition of the third vaccination dose, many Lebanese citizens have undergone this booster dose. In this context, further national studies are needed to follow the immune response after 3 vaccination doses over a longer period and to evaluate the vaccine’s effectiveness over time. The comparison of the immune response generated after previous COVID-19 infection and that generated by vaccination is also needed to make a clearer view of the immune status of the population in Lebanon.
